# Detection of SARS-CoV-2 Variants Imported Through Land Borders at the Height of the COVID-19 Pandemic in Ghana, 2022

**DOI:** 10.7759/cureus.68220

**Published:** 2024-08-30

**Authors:** Ivy A Asante, Charles N Lwanga, Cecilia Takyi, Ama N Sekyi-Yorke, Joseph A Quarcoo, Magdalene A Odikro, Emma E Kploanyi, Irene O Donkor, Adolphina Addo–Lartey, Nyarko A Duah, Daniel A Odumang, Elvis S Lomotey, Linda Boatemaa, Lorreta Kwasah, Stephen O Nyarko, Yvonne Affram, Franklin Asiedu-Bekoe, Ernest Kenu

**Affiliations:** 1 Department of Virology, Noguchi Memorial Institute for Medical Research, University of Ghana, Accra, GHA; 2 Department of Epidemiology and Disease Control, School of Public Health, University of Ghana, Accra, GHA; 3 Department of Epidemiology, School of Public Health, University of Ghana, Accra, GHA; 4 Department of Epidemiology, Noguchi Memorial Institute for Medical Research, University of Ghana, Accra, GHA; 5 Department of Microbial Pathogenesis and Immunology, Texas A&M University, College Station, USA; 6 Public Health Division, Ghana Health Service, Accra, GHA

**Keywords:** land borders, phylogeny, ghana, variants, sars-cov-2

## Abstract

Background

The World Health Organization recommends surveillance of severe acute respiratory syndrome coronavirus 2 (SARS-CoV-2) at points of entry to systematically collect and analyze data to inform decisions about the effective and appropriate use of resources needed for interventions. This study sought to determine the prevalence of SARS-CoV-2 and its variants imported into Ghana by travelers entering the country via land borders from February to July 2022.

Methods

A cross-sectional approach was employed, where recruited participants consented to the collection of oropharyngeal and nasopharyngeal samples. Specimens were analyzed for the presence of SARS-CoV-2 ribonucleic acid (RNA) using a commercially available VeriQ nCoV-OM COVID-19 Multiplex Detection kit. Amplicon sequencing protocols (ARTIC network, Oxford Nanopore Technologies (ONT), New England Biolabs, British Columbia Centre for Disease Control (BCCDC), COVID-19 Genomics UK (COG-UK), Canadian COVID-19 Genomics Network (CanCOGen), and ONT MinION) were used for SARS-CoV-2 sequencing. Logistic regression and phylogenetic analyses were conducted on the generated data.

Results

We detected a SARS-CoV-2 prevalence of 3.6% (170/4,621) among a total of 4,621 travelers screened. The average age of travelers was 32.11 ± 11.77, with the majority being male (68%, 3,132/4,621). After adjusting for educational status, household size, vaccination status, and study site, those with primary and tertiary education levels had 1.74 (95% CI: 1.16-2.62, P = 0.007) and 2.27 (95% CI: 1.27-4.05, P = 0.006) higher odds of testing positive for SARS-CoV-2 compared to those with no education. Vaccinated travelers had 0.65 odds (95% CI: 0.48-0.89, P = 0.007) of testing positive for SARS-CoV-2. The Omicron variant (B.1.1.529) emerged as the predominant lineage, constituting 77% (27/35) of isolates, compared to Alpha, Delta, and Recombinant variants. Phylogenetic analysis corroborated this finding, highlighting Delta and Omicron as the dominant circulating SARS-CoV-2 variants. Notably, Ghanaian strains from this study clustered with global variants, suggesting multiple introductions, likely through land borders.

Conclusion

A low prevalence of SARS-CoV-2 was recorded in this study, prompting the decision to reopen land borders and ease pandemic-related travel restrictions. Omicron was identified as the dominant variant. These findings emphasize the crucial role of routine surveillance at port health and advocate for a collaborative approach to addressing public health crises, preventing unnecessary travel and trade restrictions through data-based decision-making.

## Introduction

Severe acute respiratory syndrome coronavirus 2 (SARS-CoV-2), the causative agent of the novel COVID-19, spread globally after its first reported and confirmed case in Wuhan, China, on December 30, 2019. SARS-CoV-2 is a novel member of the coronavirus family [1]. Compared with SARS-CoV and Middle East respiratory syndrome coronavirus (MERS-CoV), SARS-CoV-2 is more pathogenic due to its constantly evolving genome [2]. The World Health Organization (WHO) declared the outbreak a public health emergency of international concern on January 30, 2020. As cases increased worldwide, the outbreak was declared a global pandemic on March 11, 2020 [3]. The importation of SARS-CoV-2 into Africa was inevitable, given the volume of air travel and the movement of tourists, traders, and workers between countries [3]. In Africa, the number of COVID-19 cases and associated deaths has been lower compared to the European, Asian, Australian, Northern, and Southern American continents [4]. Over 8 million COVID-19 cases and more than 170,000 deaths have been recorded on the African continent, accounting for 1.4% and 2.6% of global cumulative COVID-19 cases and deaths, respectively [5].

Ghana’s Ministry of Health reported that the first cases of SARS-CoV-2 in March 2020 were among international passengers who had traveled into the country from Turkey and Norway [6]. Response strategies were implemented in Ghana to curb and monitor the spread of COVID-19 since its first confirmed cases. These strategies included immediate measures to detect, contain, and prevent the spread of the disease. They involved the closure of land, sea, and air borders, a ban on public gatherings, and a partial lockdown of Ghana’s two major cities: Greater Accra and Greater Kumasi [7, 8]. According to the Ghana Health Service (GHS), more than 80% of COVID-19 cases in Ghana were asymptomatic, and only severe cases were admitted to health facilities [2]. In the early days of the pandemic, the Noguchi Memorial Institute for Medical Research (NMIMR) was the only testing facility ready to test as of December 2019. The NMIMR was later joined by the Kumasi Collaborative Institute for Research early in the pandemic. By May 2020, the National Public Health Reference Laboratories and the Veterinary Services Directorate laboratories in Accra were trained to support testing. By September 2020, private laboratories were trained and began testing as well. As testing capacity increased, Ghana’s COVID-19 numbers grew steadily from over 17,000 within three months of the first confirmed cases to over 150,000 confirmed cases and 1,300 deaths by January 2022. As of April 7, 2024, over 170,000 cases and 1,400 deaths have been recorded [7, 9, 10]. The COVID-19 numbers recorded, and the response strategies implemented, heavily burdened Ghana’s already strained healthcare system [11, 12].

**Figure 1:**
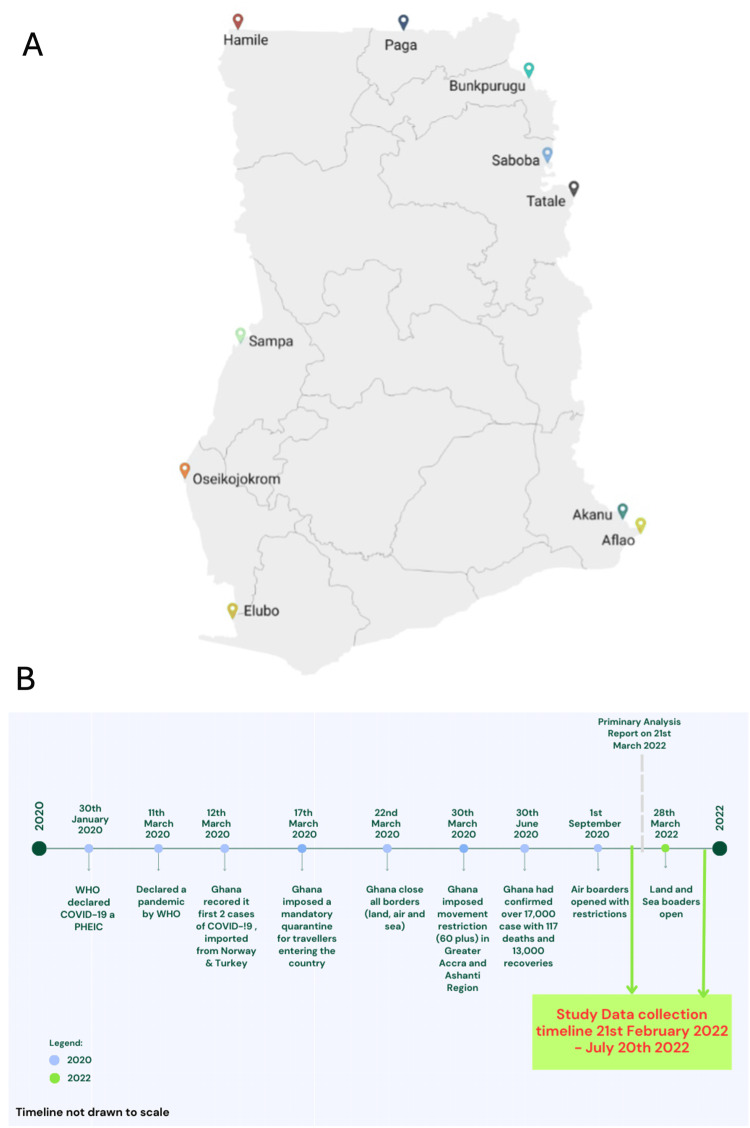
Map of Ghana showing points of entry (PoE) included in this study and the evolution of the COVID-19 pandemic in Ghana, along with the present study timeline.

Following the closure of Ghana’s borders on March 22, 2020, the country's international airport reopened on September 1, 2020, with COVID-19 screening for all travelers [8]. Ghana, as a member of the African Continental Free Trade Area (AfCFTA), signed the Free Movement Protocol at the 2018 Kigali Summit to establish a visa-free zone and pave the way for a liberalized market for goods and services within AfCFTA countries, which include neighboring countries [13]. Traffic from land borders in Ghana remained significant due to Ghana’s key role in regional trade connectivity within Africa and West Africa. Ghana’s trade has been concentrated on a few products for export, with trade to other African countries estimated at $2 billion in 2017. Intra-Africa exports accounted for 14% of global exports. The bulk of these exports were destined for other countries in the ECOWAS region, with top destinations being Burkina Faso, Togo, Niger, and Senegal [14]. Therefore, although land borders were closed, they remained accessible to cargo and truck drivers at the start of this study in February until March 28, 2022, when the land borders were fully opened, allowing for the enrollment of the general public utilizing the borders (Figure 1) [15].

Previous genomic surveillance studies from passengers using flight routes into Ghana indicated the importation and circulation of variants [16, 17]. Such findings necessitated the need to investigate the importation of SARS-CoV-2 variants through land borders as well. It was important to determine the influx of new viral strains through other borders, to facilitate the preparedness of the health task force, and to assess the need for vaccination and public education, especially among travelers. This study describes the importation of COVID-19 into Ghana through land borders and the diversity of SARS-CoV-2 variants detected through local sequencing efforts.

## Materials and methods

Study design

We conducted a cross-sectional study among adults 18 years and above entering Ghana via 10 major land borders. Participants were interviewed using a semi-structured questionnaire; nasopharyngeal and oropharyngeal swabs and blood samples were collected for laboratory diagnosis. Through RT-qPCR, the prevalence of active SARS-CoV-2 infection was identified. Sequencing was performed to identify imported variants.

Study setting

This study was conducted at 10 land borders across Ghana. Ghana is a West African country with an estimated 31 million people, 16 administrative regions, and 261 districts [18]. The country shares boundaries with Togo to the east, Côte d'Ivoire to the west, Burkina Faso to the north, and the Gulf of Guinea to the south (Figure 1). This geographic position allows for constant interaction between Ghanaians and travelers from these neighboring countries. Ghana has recognized land borders through which it interacts with its neighbors. These points of entry include Paga and Hamile, which share a border with Burkina Faso; Sampa, Elubo, and Oseikojokrom, which share a border with Côte d’Ivoire; and Akanu, Aflao, Tatale, Bunkpurugu, and Saboba, which share a border with Togo. The land borders were fully opened for use in March 2022 after implementing stringent measures to prevent the import of SARS-CoV-2 (Figure 1).

Study population

The study included individuals 18 years and above who entered Ghana at one of the 10 selected entry points. The study excluded persons 18 years and above who were border residents.

Sample size determination

The sample size was estimated using the formula n = Z² × p(1 - p)/E². Using an assumed 50% seroprevalence (p), a 95% confidence interval, and a 5% margin of error (E), a sample size (n) of 384 participants was estimated for each point of entry (PoE). A sample size of 384 individuals was estimated for each of the 10 PoEs, giving a combined minimum sample size of 3,840.

Sampling technique and data collection

Adult travelers who entered the nation through the 10 PoEs were randomly enlisted to participate in this study after giving their consent. At the time of enrollment, participants were administered a semi-structured questionnaire in person to obtain their demographic information. Data on vaccination status were also collected. Nasopharyngeal and oropharyngeal swabs were collected from everyone to test for SARS-CoV-2 infection using rapid diagnostic antigen tests followed by polymerase chain reaction (PCR) at the Noguchi Memorial Institute for Medical Research (NMIMR). Travelers who tested positive by rapid diagnostic test (RDT) were referred to healthcare facilities for further management.

Laboratory sample collection and processing

Nasopharyngeal and oropharyngeal swab samples collected from travelers entering the country through any of the points of entry were transported to the NMIMR under cold chain (4ºC) in viral transport media (VTM) for testing for the presence of SARS-CoV-2. QIAamp viral RNA extraction kit (Qiagen) was used to isolate viral ribonucleic acid (vRNA) from samples. The presence of SARS-CoV-2 was detected using the VeriQ nCoV-OM COVID-19 Multiplex Detection kit (Cat. No. 7K105). This was done using a one-step rRT-PCR targeting the SARS-CoV-2 N gene and ORF3a gene. A sample was considered conclusively positive in valid tests if there was an amplification curve observed for both the N and ORF3a genes or the ORF3a gene only, crossing the cut-off cycle threshold at Ct < 40. Otherwise, it was considered negative. Quality control was performed using Positive Control (PC) and Internal Positive Control (IPC). The validity of the test was accepted if an amplification curve was observed for PC and IPC.

Genomic sequencing and analysis

The ARTIC amplicon sequencing protocol (ARTIC network, Oxford Nanopore Technologies (ONT), New England Biolabs, British Columbia Centre for Disease Control (BCCDC), COVID-19 Genomics UK (COG-UK), Canadian COVID-19 Genomics Network (CanCOGen), and Oxford Nanopore Technologies (ONT) MinION) was used for SARS-CoV-2 sequencing. We modified the manufacturer’s protocol of the QIAamp viral RNA extraction kit to use 280 µL of transport medium from nasopharyngeal and oropharyngeal swabs collected to isolate RNA, with subsequent elution in 30 µL of elution buffer. We synthesized complementary DNA using Lunascript RT (New England Biolabs Inc., Ipswich, Massachusetts), and 400 bp amplicons were generated using the ARTIC nCoV-2019/V3 primer sets (Josh Quick, 2020). Products were quantified using the Qubit™ dsDNA HS Assay Kit (Thermo-Fisher Scientific, Waltham, Massachusetts), and DNA concentrations were normalized to 15 ng/µL for further use. Amplicons were subjected to end repair using the Ultra II End Prep (New England Biolabs Inc.). DNA fragments were barcoded using the NBXX Barcode kit (Oxford Nanopore Technologies, Oxford, UK), after which a clean-up was performed using Agencourt AMPure XP (Beckman Coulter Inc., TX, USA) beads. Adapters were then added using Adapter Mix (AMII) (Oxford Nanopore Technologies) with Quick T4 DNA Ligase (New England Biolabs Inc.). This was cleaned using Agencourt AMPure XP (Beckman Coulter Inc., Brea, California) beads and loaded onto the MinION. Lineages were typed using Pangolin (v 3.1.17) and PangoLEARN (06/12/2021). Sequences with >50% coverage were deposited in GISAID.

Data analysis

Data were downloaded in the Excel format (Microsoft Corporation, Redmond, Washington) from Google Forms, cleaned in Excel, and exported to Stata I/C 16 for analysis (Stata Corp LLC, Texas). Categorical variables such as sex, marital status, occupation, point of entry, educational level, vaccination status, and serostatus were summarized into frequencies and proportions. Point estimates with corresponding 95% CI were determined for the prevalence of SARS-CoV-2 among travelers. The positivity rate of SARS-CoV-2 in each of the study sites was determined using the formula (confirmed cases/total cases tested) × 100%. The Molecular Evolutionary Genetics Analysis (MEGA) software (version 11, 64-bit, Philadelphia, Pennsylvania) was used to generate a phylogenetic analysis. Pearson’s chi-square test of association was conducted to determine the relationship between the independent variables and SARS-CoV-2 positivity. Factors that were significant at a 10% significance level were selected for an adjusted logistic model. At the multivariate level, statistical significance of associations was determined at a 5% significance level. A crude binary logistic regression analysis was performed to assess factors associated with SARS-CoV-2 positivity among travelers. Additionally, multiple logistic regression analysis was performed to control for the effect of other variables.

## Results

Prevalence of SARS-CoV-2 and demographic characteristics of travelers

The study actively recruited 4,621 participants, recording a 3.7% (170/4,621) (95% CI: 0.03-0.43) prevalence for SARS-CoV-2 by RT-PCR. About 68% (3,132/4,621) were males, with an average age of 32.11 ± 11.77. The age group 46 to 60 years, though the second lowest in number (11%; 494/4,621), recorded the highest proportion (5%; 14/229) testing positive for SARS-CoV-2. Those with no formal education were the most recruited (38%; 1,749/4,621) but recorded the lowest proportion of positivity (3%; 48/1,749) for SARS-CoV-2, whereas those with tertiary education recorded the highest percentage of positivity (6%; 14/229). The majority of study participants were from households with two to four individuals and recorded the highest positivity (4%; 77/503). Of the 10 PoEs, the highest number of travelers were recruited from Oseikojokrom (14%; 662/4,621). About 49% of travelers self-reported being vaccinated, of which 66 (2.9%; 66/2,268) were confirmed positive for SARS-CoV-2 using rRT-PCR. Of the unvaccinated travelers, 104 of 2,353 were SARS-CoV-2 positive by rRT-PCR (4.4%; 104/2,353) (Table 1). Household size, educational status, vaccination status, and study sites were associated with SARS-CoV-2 positivity (p < 0.05).

**Table 1:** Demographic characteristics of the travelers recruited at the entry points by positivity status, Ghana, 2022. *Statistically significant. JHS: junior high school, SHS: senior high school, Tech: technical, Voc: vocational.

Characteristics	Total (N = 4621)	Positive (n=170), n (%)	Negative (n=4449), n (%)	P-value
Sex				
Male	3132	119 (3.8)	3012 (96.2)	0.529
Female	1489	51 (3.4)	1437 (96.5)	
Age group				
18–25 years	1674	60 (3.6)	1613 (96.4)	0.730
26–35 years	1472	44 (3.0)	1427 (96.9)	
36–45 years	837	35 (4.2)	802 (95.8)	
46–60 years	504	25 (5.0)	479 (95.0)	
>60 years	134	6 (4.5)	128 (95.5)	
Educational status				
No formal education	1749	48 (2.7)	1699 (97.1)	0.026*
Primary	912	42 (4.6)	870 (95.4)	
JHS/middle school	1153	47 (4.1)	1106 (95.9)	
SHS/A level/Tech/Voc.	578	19 (3.3)	559 (96.7)	
Tertiary	229	14 (6.1)	215 (93.9)	
Household size				
1	419	13 (3.1)	405 (96.7)	0.029*
2–4	1912	77 (4.0)	1835 (96.0)	
5–9	1787	70 (3.9)	1717 (96.1)	
10+	503	10 (1.9)	493 (98.1)	
Vaccine status				
Vaccinated	2268	66 (2.9)	2202 (97.1)	0.006*
Unvaccinated	2353	104 (4.4)	2249 (95.6)	
Study Site				
Aflao	560	39 (6.9)	521 (93.1)	<0.001*
Akanu	481	7 (1.5)	474 (98.5)	
Bunkpurugu	434	11 (2.5)	423 (97.5)	
Elubo	619	53 (8.6)	566 (91.4)	
Hamile	96	3 (3.1)	93 (96.9)	
Oseikojokrom	662	13 (2.0)	649 (98.0)	
Paga	455	14 (3.1)	441 (96.7)	
Saboba	436	9 (2.1)	427 (97.9)	
Sampa	392	7 (1.8)	385 (98.2)	
Tatale	486	14 (2.9)	472 (97.1)	

Association between SARS-CoV-2 infection and selected traveler factors

After adjusting for educational status, household size, vaccination status, and study site, the odds of testing positive for SARS-CoV-2 were 1.74 (95% CI: 1.16-2.62, p = 0.007), 1.51 (95% CI: 1.01-2.25, p = 0.041), and 2.27 (95% CI: 1.27-4.05, p = 0.006) times higher among travelers with primary, junior high school (JHS)/middle school/SHS/A level/technical/vocational, and tertiary education levels, respectively, compared to those who had no education. Additionally, vaccinated travelers were found to have 35% reduced odds of testing positive for SARS-CoV-2 compared to unvaccinated travelers. Across all 10 study sites, there were significantly reduced odds of SARS-CoV-2 detection compared to the Aflao border, except for the Elubo and Hamile borders (Table 2).

**Table 2:** Association between SARS-CoV-2 infection and selected traveler factors, Ghana, 2022. *One-tail significant. **Two-tail significant. Significant values (p < 0.05) in bold. COR: crude odds ratio, AOR: adjusted odds ratio, CI: confidence interval, JHS: junior high school, SHS: senior high school, Tech: technical, Voc: vocational.

Variable	SARS-CoV-2 (+)	SARS-CoV-2 (-)	COR, 95% CI	P-value	AOR, 95% CI	P-value
Educational status						
No formal education	48	1699	ref.	-	ref.	-
Primary	42	912	1.79 (1.17–2.72)	0.007**	1.74 (1.16–2.62)	0.007**
JHS/middle school	47	1153	1.54 (1.02–2.32)	0.041**	1.51 (1.01–2.25)	0.041**
SHS/A level/Tech/Voc.	19	578	1.23 (0.72–2.11)	0.456	1.22 (0.72–2.06)	0.455
Tertiary	14	229	2.35 (1.27–4.34)	0.006**	2.27 (1.27–4.05)	0.006**
Household size						
1	13	405	ref.		ref.	
2–4	77	1835	1.39 (0.75–2.58)	0.293	1.37 (0.75–2.50)	0.295
5–9	70	1717	1.65 (0.87–3.13)	0.12	1.62 (0.87–3.00)	0.122
10+	10	493	0.95 (0.52–1.74)	0.871	0.95 (0.53–1.71)	0.871
Vaccination status						
Unvaccinated	104	2247	ref.	-	ref.	-
Vaccinated	66	2202	0.64 (0.47–0.88)	0.007**	0.65 (0.48–0.89)	0.007**
Study site						
Aflao	39	521	ref	-	ref	-
Akanu	7	474	0.19 (0.09–0.44)	0.000*	0.20 (0.09–0.46)	0.000**
Bunkpurugu	11	423	0.34 (0.17–0.68)	0.002**	0.36 (0.18–0,70)	0.003**
Elubo	53	566	1.25 (0.81–1.92)	0.308	1.22 (0.82–1.82)	0.308
Hamile	3	93	0.43 (0.13–1.42)	0.167	0.44 (0.24–0.80)	0.174
Oseikojokrom	13	649	0.26 (0.14–0.50)	0.000**	0.28 (0.15–0.52)	0.000**
Paga	14	441	0.42 (0.22–0.79)	0.007**	0.44 (0.24–0.80)	0.008**
Saboba	9	427	0.28 (0.13–0.58)	0.001**	0.29 (0.14–0.60)	0.001**
Sampa	7	385	0.24 (0.10–0.55)	0.001**	0.25 (0.11–0.56)	0.001**
Tatale	14	472	0.39 (0.05–0.10)	0.004**	0.41 (0.22–0.75)	0.004**

SARS-CoV-2 variants identified among travelers at selected PoE in Ghana

**Figure 2:**
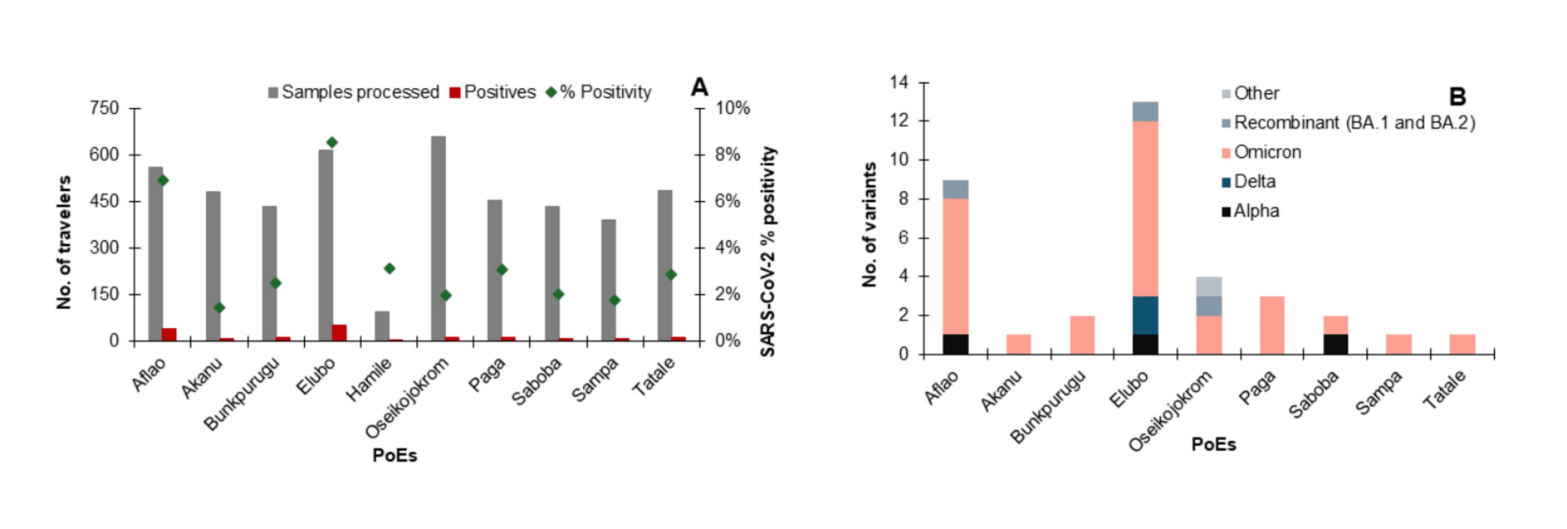
SARS-CoV-2 detection at ten entry points (PoE) across the country. Graph (A) illustrates the number of travelers recruited, the number of positive cases detected, and the percentage positivity for each entry point. Graph (B) shows the variants identified at each entry point, indicating the most and least detected variants.

Of the 10 PoE sites, Elubo registered the highest positivity rate of 9% (53/619) for SARS-CoV-2, followed by Aflao with 7% (39/560), with Akanu recording the lowest at 1% (7/481) (Figure 2A). Of the 170 rRT-PCR-confirmed SARS-CoV-2 positive cases, 36 were successfully sequenced; approximately 78% (28/36) were Omicron variants, which were identified across all entry points. Alpha and the recombinant variants were each detected at three entry points (Figure 2B).

Phylogenetic analysis of SARS-CoV-2 variants

**Figure 3:**
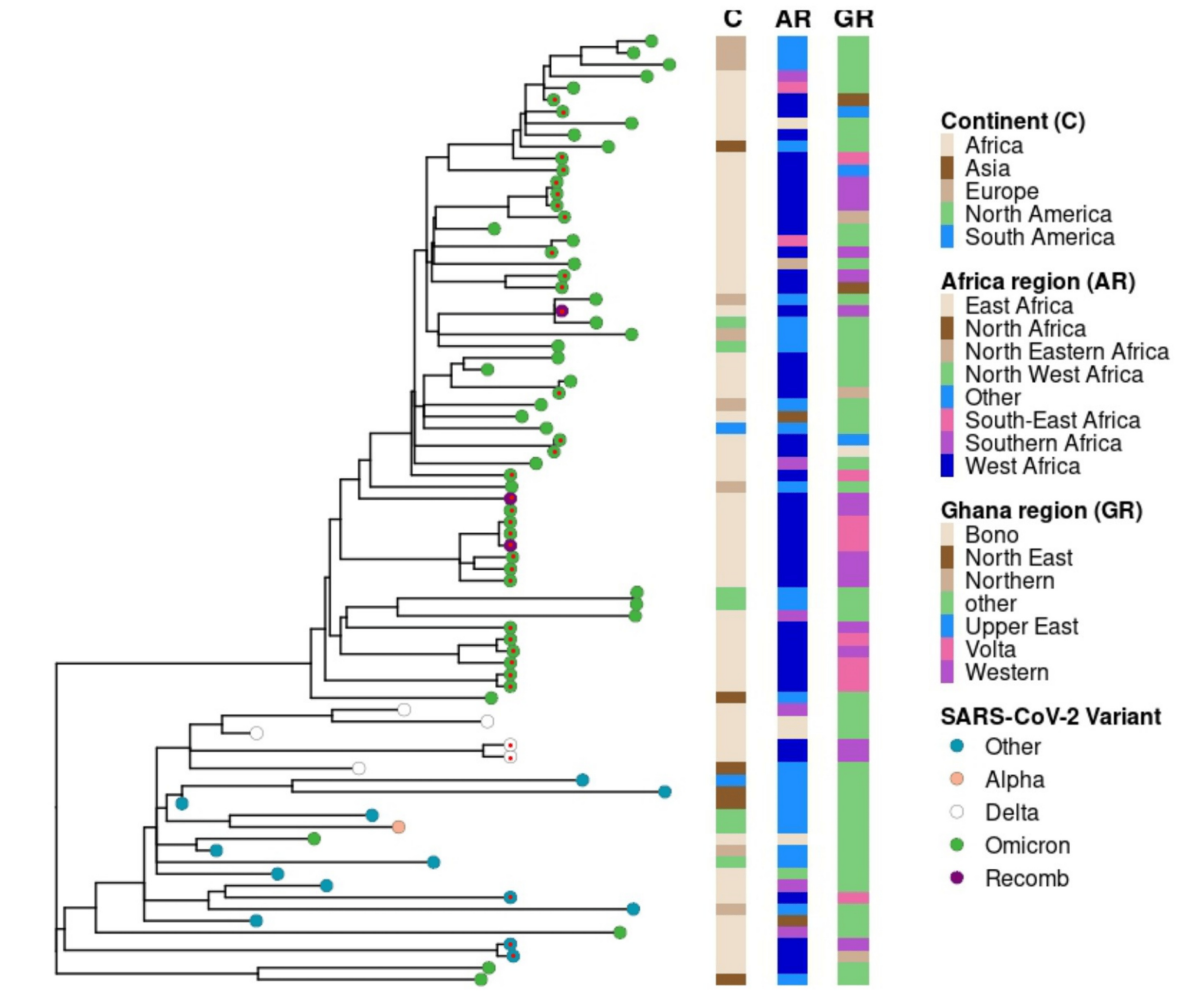
Phylogenetic relationships among Ghana variants compared to other countries. Additional sequences were retrieved from public databases, including GISAID (https://gisaid.org/resources/commentary-on-gisaid/) and GenBank. Maximum likelihood phylogenetic analysis using TreeTime and R software was performed to construct a tree depicting the evolutionary relationships among different waves of SARS-CoV-2 variants. Bootstrap values greater than 70% of 1000 replicates are shown at the nodes. The lineages of the viral samples are represented by the rings in the tree. A total of 46 global sequences were incorporated into this study.

Phylogenetic analysis showed that strains from Ghana clustered with global variants, indicating the possibility of multiple introductions (Figure 3). Our data revealed that the Omicron variant (B.1.1.529) exhibited the highest prevalence and was geographically widespread, detected in all regions of Ghana. Conversely, Delta variant strains (B.1.617) identified in this study were restricted to the Western region, suggesting a more localized presence. The predominance of the Omicron variant during this period was further supported by the identification of four distinct Omicron sub-lineages (BA.1 to BA.5).

## Discussion

In this study, we confirmed the importation of SARS-CoV-2 across land borders, its prevalence across the 10 land entry points, and the prevalence of variants detected during the study period.

Since the first SARS-CoV-2 case was detected in Ghana, the national prevalence has been estimated at approximately 7% [19]. In this study, we observed a prevalence of 3.7% (170/4,621) among travelers entering Ghana using approved land borders between February and July 2022. The lower comparative prevalence in this study may be partially attributed to the study period. Generally, from March to May 2022, cases were low in Ghana and other neighboring countries (Togo, Côte d’Ivoire, Benin, Burkina Faso, and Nigeria) [20]. Additionally, the inclusion criterion for this study was 18 years and above, which may have impacted the lower detection rates. A similar study in Nepal, with a higher prevalence of 6%, had no age cap for the participants recruited into the study [21]. Furthermore, the strict implementation of nonpharmaceutical interventions at the borders may have limited transmission, reducing detection rates among the population entering Ghana through the borders [22].

The age group 46 to 60 years, though the second lowest in number, recorded the highest proportion testing positive for SARS-CoV-2 compared to the other age groups in this study. Baynouna et al., in a case-control study aimed at assessing the risk of COVID-19 disease and estimating its prevalence in the Abu Dhabi population, concluded that age is a consistent factor for SARS-CoV-2 infection and severity [23]. Studies indicate that susceptibility to SARS-CoV-2 infection escalates with age, primarily due to weakened immunity, underlying health conditions, and decreased mobility [24, 25]. This observation aligns with our findings, as we detected higher SARS-CoV-2 positivity in our older population (46 to 60 years). However, seroprevalence investigations for SARS-CoV-2 have reported that individuals under 35 years old exhibit the highest seroprevalence rates [26, 27]. Additionally, this younger age group, unless immunocompromised, is more likely to be asymptomatic, thus escaping isolation while shedding the virus [28].

Viral transmission is influenced by contact patterns, environmental, and socioeconomic factors [29]. An interesting finding from this study was that people with no formal education recorded the lowest positivity. Studies have shown that SARS-CoV-2 positivity rates are strongly associated with low socioeconomic status [30, 31]. Allan-Blitz et al. (2021) identified that residents of Los Angeles with lower average annual household incomes, lower rates of employment, or lower rates of health insurance were more likely to be associated with SARS-CoV-2 positivity [31]. In Ghana, people with tertiary education are associated with higher income rates compared to those with no formal education. The fact that higher SARS-CoV-2 positivity rates were associated with educated travelers contrasts with observations elsewhere. A study conducted by Cajar et al. (2020) found that engaging in social contacts such as attending large events, certain behavioral factors like visiting gyms or bars, and occupational risks such as working in healthcare, social care, and educational institutions were associated with a higher rate of SARS-CoV-2 transmission and infection [32]. Therefore, a potential explanation could be related to behavioral patterns and social dynamics associated with persons with higher education. It is possible that persons with tertiary education engage in activities or have lifestyles that place them at greater risk of exposure to the virus in Ghana.

Since the emergence of COVID-19, tireless efforts have been made toward vaccine development to control the pandemic. Academia, industry, and governments worldwide collaborated to make effective vaccines available at an unprecedented speed until they came to fruition. Despite these incredible achievements, emerging problems of viral variants with increased transmissibility and immune escape threaten disease control alongside waning immunity over time in vaccinated individuals [33]. This study unveiled a significant association between vaccination status and SARS-CoV-2 infection among travelers, indicating that unvaccinated individuals were more likely to be affected by SARS-CoV-2. A review of COVID-19 vaccine effectiveness studies by Chi et al. (2022) confirmed this finding and highlighted the importance of taking a second dose or booster after an initial dose, as it is highly effective against COVID-19-related hospitalization and Intensive Care Unit admission [33]. This suggests that a vaccinated person may still get sick from SARS-CoV-2, indicating that the COVID-19 vaccine may be less effective in preventing disease. However, a study by Fisman et al. showed that random mixing of vaccinated populations with unvaccinated populations reduced the risk of SARS-CoV-2 infection among unvaccinated people by serving as a buffer to transmission [34]. The number of unvaccinated travelers was 85 times greater than vaccinated travelers in this study. This disparity could be due to limited access to vaccines or hesitancy toward vaccines. While vaccinations have brought relief, their global distribution has been a challenge due to inequity [35]. By December 31, 2021, of the 8 billion COVID-19 vaccine doses that were administered globally, only about 250 million (3%) had been administered in Africa [36]. In addition to the challenge of unequal access to vaccines, there is also the issue of vaccine hesitancy in Africa. Misinformation, coupled with the accelerated development of vaccines and rapid clinical trials and regulatory approval processes, has sparked anxiety and undermined people's confidence in their efficacy [37]. Amidst the spread of misinformation via various media channels and the internet, a significant number of individuals in Africa tend to harbor the belief that they will be used as subjects for experimentation with Western vaccines [38]. Although misconceptions and vaccine hesitancy surrounding vaccination have proven to be challenges, our study highlights the benefit of vaccination against SARS-CoV-2.

Following the detection of SARS-CoV-2, 10 major variants have evolved from the parent strain [39], three of which were detected in this study: Alpha, Delta, and Omicron. We predominantly observed Omicron variants during the study period. With over 30 mutations in its spike proteins, Omicron surpasses Alpha and Delta in mutational load [40]. Scientists have found that, unlike most SARS-CoV-2 variants that depend on TMPRSS2 for infecting cells, Omicron tends to efficiently use a TMPRSS2-independent endosomal route of entry, making it more capable of infecting many low TMPRSS2-expressing cells in the upper respiratory tract [41]. This localization facilitates rapid transmission through respiratory secretions. Furthermore, this alternative infection pathway may contribute to the clinical finding that Omicron typically causes milder illness, as it preferentially infects and replicates in the upper airways above the lungs and does not infect TMPRSS2-rich lung cells compared to other variants [42-47]. In terms of disease severity, the Omicron variant is less severe than the Delta variant, with a reduced risk of hospitalization by approximately 41%. Although existing COVID-19 vaccines exhibit diminished efficacy against Omicron, the immune response they generate still protects against severe forms of the disease, potentially preventing hospitalization even if infection occurs [48]. The Omicron variant of SARS-CoV-2 was first detected in Ghana during the 4th wave, which lasted from December 2021 to February 2022. The first case of Omicron in the current study was recorded in February 2022 [48]. Variations of the Omicron variant largely caused the 5th wave, which occurred in Ghana from May to July 2022. It is entirely possible that asymptomatic travelers could have served as a source for community transmission, particularly among individuals who were not included in this study due to ineligibility (below 18 years old).

This study was not without limitations. One notable constraint was the restricted pool of foreign nationals willing to participate. To mitigate this inadvertent selection bias, the study team proactively tackled the issue by intensifying public education efforts to dispel misconceptions among foreigners regarding specimen collection and SARS-CoV-2 testing. Additionally, the exclusion of those below 18 years of age may have led to lower detection rates, hence the lower prevalence of SARS-CoV-2.

## Conclusions

A low prevalence of SARS-CoV-2 was recorded in this study, prompting the decision to reopen land borders and ease pandemic-related travel restrictions. Omicron was identified as the dominant variant due to its high transmissibility. This finding emphasizes the crucial role of port health and advocates for a collaborative approach to addressing public health crises. Capacity building and preparedness plans at entry points are necessary for effective response measures without imposing unnecessary travel and trade restrictions. Continued education on the importance of vaccination is also imperative to boost compliance and dispel misconceptions surrounding vaccinations.
